# Algal Cultivation for Obtaining High-Value Products

**DOI:** 10.3390/md23030107

**Published:** 2025-02-28

**Authors:** Cecilia Faraloni, Eleftherios Touloupakis

**Affiliations:** 1 Istituto per la Bioeconomia, Consiglio Nazionale delle Ricerche, Via Madonna del Piano 10, Sesto Fiorentino, 50019 Firenze, Italy; 2 Istituto di Ricerca sugli Ecosistemi Terrestri, Consiglio Nazionale delle Ricerche, Via Madonna del Piano 10, Sesto Fiorentino, 50019 Firenze, Italy; eleftherios.touloupakis@cnr.it

Interest in renewable biomass sources has increased due to global population growth, the growing need for sustainable resources, and a surge in consumer demand for natural ingredients driven by concerns regarding the harmful effects of synthetic chemicals, leading to a rise in the use of high-value products from natural sources in the fields of human health, food, cosmetics, and animal nutrition. Microalgae are considered an attractive solution to this problem because of their photosynthetic efficiency, the diversity of their metabolic pathways, and their ability to thrive in harsh conditions.

Over the last 50 years, scientists and engineers have paid great attention to microalgae due to the increasing demand for sustainable development [1]. By using sunlight for conversion into valuable bioproducts, microalgae can contribute significantly to creating links between the water, energy, food, and climate cycles [2]. Microalgae can withstand harsh conditions in natural environments and grow in a variety of water sources [3]. Several species have attracted much attention due to their high growth rate, high capacity for CO_2_ sequestration, and lack of need for arable land [2,4,5,6].

Microalgae have evolved different strategies to survive under complex and extreme environmental conditions (high light, high salinity, extreme temperatures, nutrient deficiency, UV radiation) by adapting their metabolism. Various species of microalgae are capable of producing a large amount of secondary metabolites such as carotenoids, polyphenols, and essential oils, which have a wide range of therapeutic properties due to their antioxidant activity [7,8,9,10,11,12]. This peculiarity mainly depends on the species, strains, genetic diversity, and/or abiotic stress. Therefore, it is becoming increasingly important to understand the biology of these microorganisms in order to make informed decisions about their use in food, feeds, pharmaceuticals, and cosmetics. Numerous studies have been carried out to increase knowledge in this field and to optimize the recovery of natural antioxidant compounds under different growing conditions and with different stress factors.

The commercialization of microalgae as feedstock for natural products requires the use of efficient cultivation systems. These microorganisms can be cultivated using a variety of methods, including open and closed systems.

## 1. Advancements in Algal Cultivation Techniques

### 1.1. Design and Optimization of Photobioreactors

Photobioreactors provide controlled environments to optimize growth conditions and maximize productivity. Recent advances include the following:

- The development of novel photobioreactor geometries that improve light penetration and mixing efficiency.
- LED lighting for the precise control of light intensity and wavelength, tailored to specific microalgae species and target products.
- Automated control systems: the implementation of advanced sensors and control systems for real-time monitoring and adjustment of parameters such as temperature, pH, dissolved oxygen, and nutrient levels.

### 1.2. Nutrient Optimization

In order to optimize the growth of microalgae, their biomass, and the productivity of their by-products, a cultivation medium tailored to the specific needs of the desired microalgae species must be selected. At the same time, research into alternative and cost-effective cultivation media is essential to overcome economic barriers and thus facilitate the scaling up and commercialization of microalgae-based products.

### 1.3. Genetic Engineering and Strain Improvement

Metabolic and genetic engineering and synthetic biology are used to enhance target product synthesis, improve growth rate and stress tolerance, and reduce feedstock costs.

### 1.4. Downstream Processing

- Cell disruption techniques: efficient cell disruption is critical for releasing intracellular products.
- Extraction and purification: developing efficient and environmentally friendly extraction and purification techniques.

## 2. Challenges and Future Prospects

Despite significant progress, several challenges in the commercialization of microalgae-derived high-value products still remain, such as high production costs, challenges to scaling up, regulatory hurdles, and public perception.

The future of algal cultivation for high-value products is promising. Continued research and development efforts in the following areas are critical:

- Developing more efficient and cost-effective cultivation systems;
- Harnessing the power of synthetic biology;
- Developing sustainable and scalable downstream processing techniques;
- Addressing regulatory challenges and promoting public awareness.

This Special Issue, entitled “Algal Cultivation for Obtaining High-Value Products”, focuses on the promotion of algae capable of producing high-value products, as well as cultivation technologies, strategies, and growth conditions that will lead to the proliferation of these compounds; techniques for the extraction and purification of these compounds and their potential applications are also explored. This Special Issue comprises ten original articles and a review. Below, we provide an overview of the research results and a review of the existing literature to help readers find suitable articles for their fields of interest. The contributions are listed in the List of Contributions.

Contribution 1 investigates the impact of salinity and brewery wastewater on the mixotrophic cultivation of *Arthrospira platensis*. This study aimed to clarify the specific effects of salinity on the production of phycocyanin by *A. platensis* under mixotrophic conditions in a continuous mode, and to evaluate the effects of seawater on biochemical composition in terms of lipids, proteins, and carbohydrates in view of its use in the food market.

In Contribution 2, Cao et al. used *Synechocystis* sp. PCC6803 as a microbial chassis for heme production. The objectives of their study were to construct a genetically engineered *Synechocystis* sp. PCC6803 with high heme production, to explore effective methods to increase its heterotrophic capacity, and to investigate the optimal glucose concentration that would promote its growth in order to increase heme production.

In Contribution 3, Yang et al. demonstrated an increase in lipid production in *Aurantiochytrium* sp. DECR-KO using a two-stage strategy.

Contribution 4 investigates the optimization of biomass and fucoxanthin production of *Isochrysis galbana,* isolated from the coast of Tadjoura (Djibouti), by testing various culture media.

Contribution 5 investigates the biological profiles of the *Chromochloris zofingiensis* mutant LUT-4 under different light intensities by linking the physiological properties and molecular characteristics to assess the potential of LUT-4 in producing lutein.

In Contribution 6, Yi et al. investigated the effects of calcium gluconate, magnesium gluconate, and bainengtai as additional supplements in a culture of *Porphyridium purpureum*.

Contribution 7 investigated the production of fucoxanthin and fatty acids in *Conticribra weissflogii*. The authors proved that the content of the active substance *C. weissflogii* can be increased by adjusting the iron concentration.

In Contribution 8, An et al. identified and analyzed an indigenous *Odontella aurita* strain isolated from the coastal waters of Sonyang-myeon, Republic of Korea. The study aimed to determine the optimal culture conditions, including temperature, salinity, irradiance, and nutrient concentration, that affect the growth of this species and to analyze the composition of fatty acids and carotenoid pigments to investigate its potential use in various industries.

Contribution 9 investigated scalable methods to produce a highly concentrated biomass of *Scenedesmus rubescens* under heterotrophic conditions. Their aim was to find an optimal culture medium for the cultivation of *S. rubescens* under heterotrophic conditions.

In Contribution 10, the authors review recent advances in genetic engineering and cultivation strategies to improve the production of lutein by microalgae. Techniques such as random mutagenesis; genetic engineering, including CRISPR technology; and multi-omics approaches are discussed in detail in terms of their impact on improving lutein production. Innovative cultivation strategies are compared, and their advantages and challenges are highlighted.

This Editorial’s concluding remarks are as follows:

Microalgae provide a sustainable and versatile platform to produce a wide range of high-value products. Recent advances in algae cultivation techniques, including the development of photobioreactors, nutrient optimization, genetic engineering, and downstream processing, are paving the way for more efficient and cost-effective production. Addressing the remaining challenges and seizing opportunities in this area will be critical to realizing the full potential of algae as a source of sustainable and valuable resources. Continued innovation and collaboration between researchers, industry, and policy makers will be crucial to driving the commercialization of high-value algae products and contributing to a bio-based economy.

We would like to thank the Editorial Board, the Managing Editors, and the Editorial Assistant. We greatly appreciate the efforts of the authors who have contributed their results to this Special Issue. Finally, we thank the reviewers who carefully evaluated the submitted manuscripts for their support.
